# Postural Responses to a Suddenly Released Pulling Force in Older Adults with Chronic Low Back Pain: An Experimental Study

**DOI:** 10.1371/journal.pone.0162187

**Published:** 2016-09-13

**Authors:** Pei-Yun Lee, Sang-I Lin, Yu-Ting Liao, Ruey-Mo Lin, Che-Chia Hsu, Kuo-Yuan Huang, Yi-Ting Chen, Yi-Ju Tsai

**Affiliations:** 1 Department of Physical Therapy, College of Medicine, National Cheng Kung University, Tainan, Taiwan; 2 Department of Orthopedic, Tainan Municipal An-Nan Hospital-China Medical University, Tainan, Taiwan; 3 Department of Orthopedic, National Cheng Kung University Hospital, Tainan, Taiwan; University of Ottawa, CANADA

## Abstract

Chronic low back pain (CLBP), one of the most common musculoskeletal conditions in older adults, might affect balance and functional independence. The purpose of this study was to investigate the postural responses to a suddenly released pulling force in older adults with and without CLBP. Thirty community-dwelling older adults with CLBP and 26 voluntary controls without CLBP were enrolled. Participants were required to stand on a force platform while, with one hand, they pulled a string that was fastened at the other end to a 2-kg or to a 4-kg force in the opposite direction at a random order. The number of times the participants lost their balance and motions of center of pressure (COP) when the string was suddenly released were recorded. The results demonstrated that although the loss of balance rates for each pulling force condition did not differ between groups, older adults with CLBP had poorer postural responses: delayed reaction, larger displacement, higher velocity, longer path length, and greater COP sway area compared to the older controls. Furthermore, both groups showed larger postural responses in the 4-kg pulling force condition. Although aging is generally believed to be associated with declining balance and postural control, these findings highlight the effect of CLBP on reactive balance when responding to an externally generated force in an older population. This study also suggests that, for older adults with CLBP, in addition to treating them for pain and disability, reactive balance evaluation and training, such as reaction and movement strategy training should be included in their interventions. Clinicians and older patients with CLBP need to be made aware of the significance of impaired reactive balance and the increased risk of falls when encountering unexpected perturbations.

## Introduction

Low back pain is a common musculoskeletal condition that causes physical disability and other burdens to individuals, society, and the economics [1–3]. Most people have low back pain sometime during their life: the 1-year prevalence is 38.1% and the estimated point prevalence is 18.1% [4]. Most people with low back pain tend to have recurrent episodes, and approximately 5–15% develop chronic low back pain (CLBP) [5,6]. As populations rapidly age, the incidence of CLBP increases remarkably. The prevalence of CLBP is highest in people between 60 and 79 years old, and is possibly underestimated in older populations [4,7]. Older age is a recognized prognostic factor for having CLBP [8]. Therefore, it is essential to investigate CLBP in the older populations.

Balance, the ability to control the body center of mass within the base of support or stability limits, is important for maintaining postural stability and preventing falls during daily activities [9,10]. Proactive balance control is the ability to modify postural control before a potentially destabilizing movement in order to avoid instability, and reactive balance control is the ability to recover postural control after an unexpected perturbation. Both control mechanisms are important for independently and safely performing functional activities and responding to environmental changes.

Some studies have reported increased postural sway during quiet standing and walking in 33- to 46-year-olds with CLBP [11–14]. Other studies have reported that 39- to 60-year-olds with CLBP have poorer balance performance during single-leg standing, timed up and go, and forward reaching tests [15,16]. Such proactive balance deficits significantly contribute to functional declines and dependence in people with CLBP. In contrast, only relatively few studies have demonstrated that 29- to 37-year-olds with CLBP had a delayed or a large center of pressure (COP) response, and made more postural adjustments while standing on a movable platform that was unexpectedly translated [17,18]. Such a reactive balance deficit might lead to more frequent falls, because falls generally occur due to an inadequate reactive response to an external force [19,20]. Pain-induced movement- limitation of the spine, reduced lumbar proprioception and sensory feedback from the lower extremity, and trunk muscle weakness and atrophy might all be related to the impaired balance in people with CLBP [21–25].

However, most previous research on CLBP has been focused on younger people, or has included single groups with wide age spectra and a mean age typically < 60. The balance performance and control of older adults with CLBP might be different from those of younger adults, and findings from widely mixed-age groups might not be applicable to older populations. Fear-avoidance beliefs are strongly associated with pain and disability in patients with CLBP, and lead to a poorer prognosis [26–28]. Older patients with CLBP might be more concerned or fearful about movements that produce pain, and thus might limit their daily activities or modify their movement strategies when they encountered postural perturbations. With a rapidly increasing older population, the number of older adults having CLBP is expected to grow. To comprehensively understand the balance problem of older adults with CLBP thus has become extremely important and should provide the rationale for developing more effective conservative interventions.

A few studies have reported that older adults with CLBP showed poor balance performance on the clinical tests of single-leg standing, five times sit-to-stand, alternative stepping, and timed up and go, or that they had an increased risk of falls [15,29,30]. However, none have ever investigated reactive balance control, especially in older adults with CLBP. Previous research has used unexpected platform translation or catching a falling object to study the postural and neuromuscular responses of younger people with CLBP [17,31]. However, these conditions are not commonly seen during everyday life. In fact, pulling an object that might suddenly move or open, such as a stuck drawer or a heavy door, is more common. The postural responses to a suddenly released pulling force remain unclear. In particular, such a pulling task would probably place a significant demand on the trunk and result in a larger challenge to the older adults with CLBP. Therefore, the main purpose of this study is to investigate the postural responses to a suddenly released pulling force in older adults with and without CLBP. It is hypothesized that, in response to two levels of suddenly released pulling forces, older adults with CLBP would have delayed, longer, and larger COP movements than would older adults without CLBP, and that for both groups, a larger pulling force would result in quicker, longer, and larger COP movements than would a small pulling force.

## Methods

### Participants

A total of 56 participants (≥ 55 years old) were enrolled (S1 Data): 30 with CLBP (CLBP group) and 26 healthy without CLBP (HEA group). They were required to have sufficient cognitive ability to follow three-step commands and perform the experimental protocol; thus, a score of Mini-Mental State Examination (MMSE) score > 24 (out of 30) was requested. The inclusion criteria for the CLBP group were as follows: (1) nonspecific low back pain for at least 3 months in the previous year with (2) the worst pain during the last three months rated ≥ 3 (out of 10) on the visual analogue scale (VAS). The VAS measures pain intensity of a patient from 0 (no pain) to 10 (most severe pain). Patients with CLBP were excluded from the study if any of the following exclusion criteria were met: it was the potential participant’s first episode of low back pain, the potential participant had a history of falls in the previous year, a history of spinal or low-extremity surgery, overt neurological signs or symptoms (severe sensory deficits or motor paralysis), spinal tumors, acute vertebral compression fracture, rheumatological diseases of the spine, symptoms of fecal and urine incontinence, or any physical injury or medical condition that might affect balance. HEA group had no history of low back pain for a minimum of 1 year, and no history of falls.

This study was approved by the Institutional Review Board of the National Cheng Kung University Hospital. Potential participants were recruited from the National Cheng Kung University Hospital, local communities, and around the campus. Eligible participants were informed about the purpose and experimental procedures of the study, and all signed an Institutional Review Board-approved informed consent form before the study began.

### Instruments

**Fig 1:**
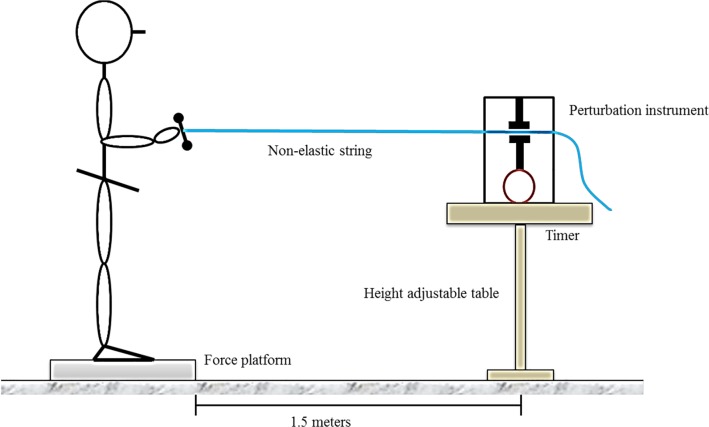
Experimental setting for postural perturbation.

Ground reaction forces were collected using a force platform (Type 5233; Kistler, Inc., Winterthur, Switzerland) embedded in the laboratory floor. Unexpected postural perturbation was created using a custom-made instrument composed primarily of a compressor that can tightly compress and fasten a non-elastic string passing through it while an individual can pull from the other end of the string (Fig 1). When the string is released by the compressor, this instrument suddenly releases the pulling force and produces a postural perturbation. A built-in timer can adjust when the string is released; thus, an unexpected perturbation can be created. All instruments, including the force platform and perturbation machine, were synchronized simultaneously using a National Instruments acquisition card (National Instruments, Austin, Texas, USA) at a sampling rate of 1000 Hz through the LabVIEW program (LabVIEW 2002, National Instruments).

### Procedures

Data were collected at the university’s Motion Analysis Laboratory. Standard experimental procedures were used: a series of questionnaires to record the participants’ basic information, pain conditions, physical activity levels, pain-related disabilities, and fear avoidance beliefs; sensorimotor assessment and postural perturbation experiment. In addition, anthropometric measurements (body height, weight, and participants’ foot length) were recorded for each subject.

### Questionnaires

The Chinese version of the International Physical Activity Questionnaire (IPAQ) was used to estimate the physical activity levels of all participants. The IPAQ consists of four categories of activities with different activity levels: vigorous, moderate, light intensity of walking, and sitting. Participants were asked to report all 10-minute activities they had done during the previous 7 days. The total metabolic-equivalent-minutes-per-week was calculated and reported. For CLBP patients, a modified Oswestry Low Back Pain Disability Questionnaire (Chinese versions) and a modified Fear-Avoidance Beliefs Questionnaire (Chinese version) were used to rate the patients’ levels of pain and disability, and their fear of movement beliefs. The Oswestry Disability Index (ODI) ranges from 0 to 100; a higher score indicates a more severe disability [32]. The FABQ measures the level of fear of physical activity (FABQ-PA) and work (FABQ-W) (maximum score: 24 and 42, respectively). A higher score of the FABQ indicates greater fear and avoidance beliefs about physical activity or work [33]. Because 20 out of the 30 participants in the CLBP group were retired, only their FABQ physical activity score were reported. The reliability and validity of each questionnaire have been confirmed [34–36].

### Sensorimotor assessment

Left-side and right-side sensorimotor function were measured, and their means were used for data analysis. Plantar touch-pressure (cutaneous) sensitivity was assessed using Semmes-Weinstein monofilaments (Patterson Medical, Warrenville, IL, USA); which consist of 20 filaments with different thicknesses of nylon string. During the testing, the filament was applied perpendicularly and slowly to the test site for 1.5 seconds until the filament bowed, and then the participant was asked to indicate whether they felt pressure. The smallest filament that the participant was able to perceive indicated their touch-pressure sensitivity. Participants were tested in the sitting position with their eyes closed to eliminate visual cues. Six sites for the plantar sides of both feet were assessed: the first and fifth metatarsal heads and the center of the heel.

Hand-grip strength was assessed using a calibrated hand dynamometer (JAMAR; Sammons Preston Rolyan, Bolingbrook, IL, USA). The participants were seated with their one arm at their side and in neutral rotation, elbow in 90° flexion, and forearm in neutral position, and then they exerted maximum volitional contraction for 3 seconds. The maximum isometric strength of the hip flexors, knee flexors, knee extensors, and ankle dorsiflexors was measured using a handheld dynamometer (MicroFET2; Hoggan Health Industries, Salt Lake City, UT, USA) using standard test procedures [37]. The grip and strength were measured bilaterally and normalized to body weight. The strength of the ankle plantar flexors was tested using manual muscle testing at a grade level of 25 [37].

### Postural perturbation experiment

Participants stood barefoot and shoulder-width apart on the force platform. After ensuring that they had adopted their natural comfortable standing position, an outline of their feet was traced to mark the starting position, to which they were required to return for every trial. The custom-made perturbation instrument was placed on a height-adjustable table that was 1.5 m in front of the force platform. Participants were required to stand naturally and use their right upper limb to randomly pull the fastening string of either the 2-kg (medium) or 4-kg (large) force at the joint positions of shoulder flexion 0°, elbow flexion 90°, and wrist extension 20° (Fig 1). The magnitude of the pulling force, determined based on our pilot study finding that 4 kg was the 50% of the mean peak force that 10 healthy older adults could pull without losing their balance, was monitored using a digital luggage scale connected to the string. Participants were instructed to maintain their balance without moving their feet when the string was suddenly released. Two trials were obtained for each pulling force. The number of losses of balance was recorded if the participant moved their foot or had a stepping reaction. They were allowed one practice trial to familiarize themselves with the procedures and testing conditions.

### Data reduction and analysis

**Fig 2:**
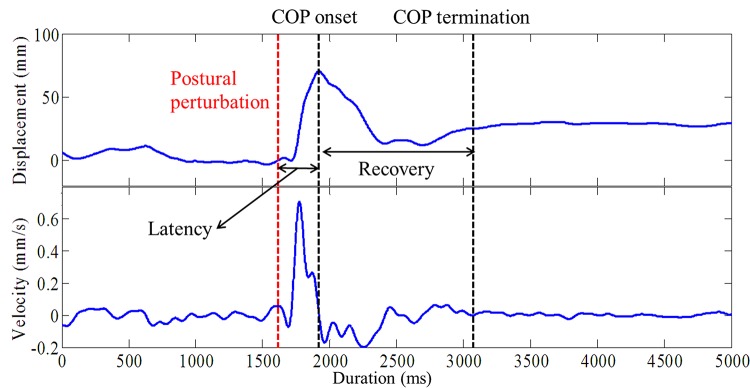
A representative example of the center of pressure (COP) position and velocity in the anterior-posterior direction when responding to a suddenly released pulling force. For both COP displacement and velocity, a positive value indicates a posterior displacement or velocity, and a negative value indicates an anterior displacement or velocity. COP onset latency is the duration between postural perturbation and COP onset. The recovery event is defined as from the COP onset to the COP termination.

All data were processed and analyzed using computer algorithms written in MATLAB 17 (MathWorks, Inc., Natick, MA, USA).COP data were calculated from the ground reaction forces and filtered using a 4th order Butterworth low-pass filter at 10Hz. To describe the characteristics of the postural reactions in different movement directions, the COP displacement and instantaneous velocity in both anterior-posterior (AP) and medial-lateral (ML) directions were calculated. The onset of COP movement was defined as the time when the COP was in its most posterior position and began to move anteriorly (Fig 2). The termination of COP movement was defined as the time point when COP velocity in the AP direction reached one standard deviation below the baseline of the velocity for 50ms. The baseline of the velocity was defined as the mean velocity from 1000 to 2000ms after data collection when participants were requested to pull the fastened string steadily before the perturbation was introduced. Several COP parameters were then calculated from the onset to the termination of COP movement. The ranges of COP displacements in both the AP and the ML directions were calculated, the peak COP displacements in both directions were identified, and the mean and peak COP velocities in both directions were also calculated. Moreover, the onset latency of the COP recovery movement was calculated from the time when the perturbation was introduced (i.e., the time point when the string was released) to the time when the COP was moved to the most posterior position and began to move anteriorly (Fig 2). The duration between the onset and termination of COP movements was also calculated. To characterize the overall movements of the COP trajectory in space, the path length and sway area (i.e., ellipse area) of the COP trajectory were also calculated from the onset to the termination of COP movements. To control for the effect of body stature and facilitate between-participant comparisons, all COP spatial variables were normalized to the participants’ foot length for statistical analysis.

### Statistical analysis

The differences in the anthropometric characteristics, physical activity levels, and muscle strength (except for ankle plantar flexor) between the two groups were compared using independent *t* tests. The between-group differences in plantar cutaneous sensation and ankle plantar flexor strength were compared using nonparametric Mann-Whitney U tests. Differences in the number of losses of balance were compared using Chi-square tests both for groups and for pulling forces. Two-way analysis of variance ANOVA with factors of group (between subjects: CLBP vs. HEA) and force condition (repeated within subjects: 2kg vs. 4kg) was used separately for the COP data. Post hoc independent or paired *t* tests were used if interactions between the group and force factors were significant. We did not apply the Bonferroni correction for multiple comparisons because this procedure is too conservative for correlated variables (i.e., motions of the COP), but the partial eta squared ($\eta_{p}^{2}$) was calculated to estimate the effect size of group or condition difference. A rule of thumb for determining the magnitude of effect size suggests that small ($\eta_{p}^{2}=0.01$), medium ($\eta_{p}^{2}=0.06$), and large ($\eta_{p}^{2}=0.14$) effects [38]. SPSS 17.0 was used for all statistical analyses. Significance was set at p < 0.05.

## Results

There were no significant differences in age, height, weight, body mass index, foot length, or level of physical activity between the CLBP and HEA groups (Table 1). Most CLBP patients complained about having had back pain for around 6.5 years; the highest VAS score for pain during the previous two weeks was 5.18. The mean ODI score for the CLBP group was 24.7%, which is considered to be at the moderate disability level (20% to 40%) [39]. The mean FABQ score for physical activity was 15.57, which indicates a high level of fear avoidance of physical activity [33].

**Table 1:** Mean (±standard deviation) of the anthropometric and back pain characteristics for the HEA and CLBP groups.

Characteristics	HEA (n = 26)	CLBP (n = 30)	p value
Age (years)	66.23 (4.53)	64.57 (5.71)	0.238
Gender ^a^	M = 15, F = 11	M = 12, F = 18	0.543
Hand dominance ^a^	R = 26, L = 0	R = 27, L = 3	0.097
Height (m)	1.62 (0.08)	1.61 (0.07)	0.787
Weight (kg)	61.59 (10.61)	65.38 (11.41)	0.211
Body mass index (kg/m^2^)	23.41 (2.80)	25.11 (3.74)	0.062
Foot length (cm)	23.64 (14.96)	23.20 (1.42)	0.271
Physical activity level (METs-min/week)	2652.54 (1527.11)	1818.76 (1571.47)	0.054
Pain duration (years)	Not Applicable	6.45 (8.44) ^b^	
Highest VAS pain intensity	Not Applicable	5.18 (2.04)	
Oswestry Diability Index (%)	Not Applicable	24.67 (13.22)	
Fear-avoidance beliefs for physical activity (/24)	Not Applicable	15.57 (5.68)	

^a^ analyzed using Chi-square test.

^b^ n = 29, one participant was missing data.

### Sensorimotor functions

There were significant group differences of plantar sensitivity only at the heel. The CLBP group had significantly poorer plantar sensitivity at heel region than the HEA group. The CLBP group also had significantly less hand-grip strength and less strength in all lower extremity muscle groups except the hip flexors (Table 2).

**Table 2:** Sensorimotor functions for the HEA and CLBP groups.

Median (range)	HEA (n = 26)	CLBP (n = 30)	*p*-value
Plantar cutaneous sensitivity (log 10)			
1^st^ metatarsal head	3.84 (3.22–4.74)	3.84 (3.22–4.56)	0.602
5^th^ metatarsal head	4.08 (2.83–4.56)	4.17 (3.22–5.07)	0.124
Heel	4.08 (3.22–5.18)	4.17 (3.61–4.56)	0.032
Strength (% Body Weight)			
Grip ^c^	47.85 (9.81)	37.69 (10.91)	0.001
Hip flexors ^c^	26.55 (7.06)	22.61 (8.05)	0.063
Knee extensors ^c^	31.21 (6.14)	26.72 (7.19)	0.018
Ankle dorsiflexors ^c^	35.73 (9.23)	28.71 (8.96)	0.007
Ankle plantar flexors (repetitions)	21.50 (0–25)	13.25 (0–25)	0.040

^c^ Presented as mean (standard deviation); statistical analysis used independent *t* tests

### Number of losses of balance

For the 2-kg pulling force, there were 2 losses of balance in both groups (CLBP: 2 in 60 trials = 3.33%; HEA: 2 in 52 trials = 3.85%). For the 4-kg pulling force, there were 17 losses of balance in the CLBP group (17 in 60 trials = 28.33%) and 9 in the HEA group (9 in 52 trials = 17.31%). Although for the 4-kg pulling force the number of losses of balance in the CLBP group was higher than that in the HEA group, no significant differences were found for the 2-kg or 4-kg pulling forces (p = 0.884 and p = 0.168, respectively).

### COP responses after perturbation

Three participants in the CLBP group and four in the HEA group fell on both trials for both pulling-force conditions. Thus, the results of COP responses were analyzed only for 23 participants in the CLBP group and 26 in the HEA group.

#### Onset latency and recovery duration of postural responses

The descriptive statistics of COP variables are summarized in Table 3. The CLBP group had a significantly longer COP onset latency than did the HEA group (F = 11.658, p = 0.001, $\eta_{p}^{2}$ = 0.199), but not a significantly different recovery duration (F = 0.356, p = 0.553). The recovery duration in response to the 4-kg force was significantly longer than that in response to the 2-kg force for both groups (F = 8.569, p = 0.005, $\eta_{p}^{2}$ = 0.154), but the onset latency was not (F = 2.724, p = 0.104). No significant interaction was found for either recovery duration or onset latency (F = 0.170, p = 0.682 and F = 1.188, p = 0.281, respectively).

**Table 3:** Mean (±standard deviation) of COP latency and duration, displacements, velocities, and trajectory movement after perturbation between medium (2kg) and large (4kg) pulling force conditions for the HEA and CLBP groups.

COP motions	HEA (n = 23)		CLBP (n = 26)	
	2kg	4kg	2kg	4kg
Temporal variables (ms)				
Duration^‡^	1723.87 (646.46)	2046.80 (717.50)	1771.91 (624.34)	2200.57 (925.79)
Onset latency^†^	387.03 (78.80)	400.89 (77.69)	443.81 (140.60)	511.10 (156.37)
Displacements in the AP direction (%FL)				
Post Peak^‡^	24.27 (10.84)	34.97 (13.84)	27.51 (9.03)	39.93 (12.26)
Range^†^^‡^	17.01 (6.82)	23.92 (10.05)	21.95 (8.25)	29.19 (8.64)
Displacements in the ML direction (%FL)				
Right Peak	4.78 (2.44)	6.37 (3.70)	7.62 8.20)	7.81 (4.28)
Range^†^^‡^	10.05 (4.05)	11.97 (5.89)	12.35 (5.17)	16.55 (10.20)
Velocities in the AP direction (%FL/sec)				
Peak^‡^	0.19 (0.09)	0.26 (0.11)	0.20 (0.07)	0.31 (0.10)
Mean^†^^‡^	0.021 (0.007)	0.023 (0.007)	0.024 (0.007)	0.031 (0.010)
Velocities in the ML direction (%FL/sec)				
Peak^‡^	0.03 (0.02)	0.06 (0.06)	0.05 (0.03)	0.10 (0.07)
Mean^‡^	0.009 (0.004)	0.011(0.004)	0.011 (0.006)	0.015 (0.010)
Trajectory movements (%FL)				
Path length^†^^‡^	6.38 (3.13)	11.05 (4.66)	9.39 (6.60)	15.78 (12.06)
Sway area^‡^	6.38 (3.13)	11.05 (4.66)	9.39 (6.60)	15.78 (12.06)

FL = foot length

^†^ p<0.05 for group main effect

^‡^ p<0.05 for force main effect

#### COP displacements

The CLBP group had a significantly greater range of COP displacements in the AP direction than the HEA group (F = 5.811, p = 0.020, $\eta_{p}^{2}$ = 0.082), but peak COP displacements in the AP direction did not differ between the two groups (F = 2.004, p = 0.163). The peak and range of COP displacements in the AP direction in response to the 4-kg pulling force were significantly greater than those in response to the 2-kg force for both groups (F = 51.705, p<0.001, $\eta_{p}^{2}$ = 0.524 and F = 34.490, p<0.001, $\eta_{p}^{2}$ = 0.423, respectively). No significant interaction was found for either peak or range of COP displacements in the AP direction (F = 0.286, p = 0.595 and F = 0.020, p = 0.899, respectively).

The CLBP group had a significantly greater range of COP displacements in the ML direction than did the HEA group (F = 4.174, p = 0.047, $\eta_{p}^{2}$ = 0.082), but peak of COP displacements in the ML direction did not significantly differ between the two groups (F = 3.430, p = 0.070). The range of COP displacements in the ML direction in response to the 4-kg force was significantly greater than that in response to the 2-kg force (F = 9.412, p = 0.004, $\eta_{p}^{2}$ = 0.167), but peak COP displacements in the ML direction were not (F = 0.931, p = 0.399). No significant interaction was found for either the peak or the range of COP displacements in the ML direction (F = 0.519, p = 0.475 and F = 1.318, p = 0.257, respectively).

#### COP velocities

The CLBP group had a significantly greater mean COP velocity in the AP direction (F = 6.890, p = 0.012, $\eta_{p}^{2}$ = 0.128) than did the HEA group, but peak COP displacements in the AP direction did not significantly differ between two groups (F = 2.201, p = 0.145). Both peak and mean COP velocities in the AP direction in response to the 4-kg pulling force were significantly greater than those in response to the 2-kg force for both groups (F = 46.677, p<0.001, $\eta_{p}^{2}$ = 0.498 and F = 18.564, p<0.001, $\eta_{p}^{2}$ = 0.283, respectively). No significant interaction was found for either peak or mean COP velocities in the AP direction (F = 2.118, p = 0.152 and F = 1.932, p = 0.171, respectively).

The CLBP group tended to have greater peak and mean COP velocities in the ML direction than did the HEA group, although did not reach the significant level (F = 3.346, p = 0.074, $\eta_{p}^{2}$ = 0.066 and F = 3.609, p = 0.064, $\eta_{p}^{2}$ = 0.071, respectively). Both peak and mean COP velocities in the ML direction in response to the 4-kg pulling force were significantly greater than those in response to the 2-kg force for both groups (F = 15.469, p<0.001, $\eta_{p}^{2}$ = 0.248 and F = 10.121, p = 0.003, $\eta_{p}^{2}$ = 0.177, respectively). No significant interaction was found for either peak or averaged COP velocities in the ML direction (F = 0.527, p = 0.471 and F = 3.308, p = 0.075, respectively).

#### COP trajectory

The CLBP group had a significantly longer path length (F = 4.587, p = 0.037, $\eta_{p}^{2}$ = 0.089) than did the HEA group. The CLBP group also tended to have a greater sway area than did the HEA group, but did not reach significant level (F = 3.895, p = 0.054, $\eta_{p}^{2}$ = 0.077). Both the path length and the sway area of COP trajectory in response to the 4-kg pulling force were significantly greater than those in response to the 2-kg force for both groups (F = 40.517, p<0.001, $\eta_{p}^{2}$ = 0.463 and F = 34.901, p<0.001, $\eta_{p}^{2}$ = 0.426, respectively). No significant interaction was found for either path length or sway area of COP trajectory (F = 0.022, p = 0.882 and F = 0.853, p = 0.360, respectively).

## Discussion

Balance is important for functional independence, especially in older people. Because the number of older adults with CLBP is increasing, it is necessary to examine their balance ability that might affect their functional capacity. The current study found that, compared with those healthy older adults, older adults with CLBP had delayed and larger postural responses (i.e., larger and faster COP movements) when responding to a suddenly released pulling force. These findings highlight the importance of reactive balance evaluation and training in older adults with CLBP.

Although we found that older adults with CLBP did not lose their balance more often than did healthy controls, their postural responses were different. The onset of COP anterior movements was delayed in older adults with CLBP compared with healthy older adults when responding to a suddenly released pulling force. After perturbation, a longer onset latency of COP anterior movements places the body in a postural unstable condition for a longer period of time. This implies an inefficient ability to regain balance and an increase in the risk of losing balance. It is commonly accepted that a delayed recovery response is an indicator of poor balance control and is associated with an increased risk of falls [31,40,41]. The reactive balance control tested in the present study inherently involves the sensory feedback process, for which the body needs to sense the postural disturbance from different body parts, and then, based on the levels of perturbation, immediately initiates the body movements required to reestablish equilibrium.

Older adults with CLBP in this study also had significantly poorer plantar sensitivity of the heel than did healthy controls, which might partly explain the delayed response in older adults with CLBP. Reduced cutaneous sensitivity in the heel region might make one unable to detect how far the COP had been passively moved to the posterior edge of the base of support, and thus might delay muscle responses and subsequent reactive responses of the body.

Other studies have reported that patients with CLBP had impaired lumbar proprioception. Such sensation deficits in the lumbar spine might also provide inaccurate information about the trunk position to the central nervous system and thus contribute to the temporal delay of COP reactive responses. Reweighting proprioceptive inputs from the trunk to the ankles has been observed in young people with low back pain, and trunk stiffening is a common strategy used while standing [42,43]. Trunk muscle atrophy and reduced strength accompanied with aging and pain might affect the ability of older adults with CLBP to adopt a trunk stiffening strategy for reactive responses. Furthermore, one study indicated that the nervous system might sense body dynamics being subjected to various types of perturbation and might choose perturbation-dependent postural feedback gains that fulfill postural stability and feasible physical constraints [44]. The perturbation paradigm used in the present study was to challenge the equilibrium system from the arm holding the fastened string to the trunk and then to the lower body; thus, to initiate balance recovery from the trunk might be a more efficient strategy in this condition. Older adults with CLBP interfered with pain and lumbar proprioceptive deficits might in turn rely more on sensory feedback and muscle activation from the lower limbs. However, these notions require additional investigations.

Delayed onset of COP anterior movements in older adults with CLBP might also lead to the COP traveling to a longer distance. The current study found that, for older adults with CLBP, the perturbation resulted in a greater range of COP displacement in the AP direction than in older adults without CLBP. A larger range of COP displacement in the AP direction implies a larger postural disturbance for older adults with CLBP; it also suggests that a larger recovery response is required to regain postural stability. The current study also confirmed that older adults with CLBP had greater postural recovery than did controls: a longer range of COP displacements in the ML direction, a longer COP traveling path length and ellipse area, and greater mean COP velocities in both directions.

In response to the suddenly released pulling forces, the COP is required to move anteriorly to regain balance, which indicates that the anterior side muscle groups e.g., the tibialis anterior, quadriceps, and abdominal muscles might have been activated. Older adults with CLBP showed greater COP movement, which might also imply larger recruitment of anterior muscles to deal with suddenly released pulling forces. The current study found that older adults with CLBP had significantly less strength in all lower extremity muscle groups except for the hip flexors, which is consistent with findings in other studies [45,46]. Thus, we hypothesize that in the present study, older adults with CLBP increased their muscle recruitment to augment muscle-force output for counteracting the demand of postural disturbance. This adaptation represents a neuromuscular disadvantage and is commonly observed in patients with muscle strength deficits and in older adults [47–49]. However, such an adaptation for older adults with CLBP might be insufficient when encountering a larger magnitude of perturbation or might deteriorate when the ability for efficiently recruiting motor units is progressively altered with aging or pain [50,51]. Electromyography should be used to investigate this in future study.

The larger range of COP displacements and faster velocities in the ML direction found in older adults with CLBP might indicate poorer postural control in the frontal plane. Many other studies have reported that a larger mediolateral COP or center of mass (COM) movement, an increased relative horizontal distance between COM and COP positions, or a greater COM and COP inclination angle in the frontal plane was indicative of impaired lateral stability and often seen in patients with balance deficits or in older adults with a history of falls [52–56]. Increased lateral movement of the body might be associated with reduced control or weak hip abductors [57]. Back pain or associated leg pain is often predominant on one side of the body, and the sensorimotor functions might be more impaired on the relatively painful leg. One study [15] found that spinal steadiness in older adults with CLBP was altered when single-leg standing on the painful leg compared to the non-painful leg indicating different movement strategies adopted and be related to the differences of sensorimotor control [15]. Patients with CLBP also showed attenuated ground reaction force imposed on their painful leg while walking [58], all of which might partly explain greater COP motion in the ML direction; however, these notions need additional investigations.

That a larger magnitude of postural perturbation induces a faster, longer, and larger recovery response is commonly accepted in the literature [59,60]. It is not surprising that our findings were similar. When responding to the 4-kg versus the 2-kg pulling force, both groups showed a longer recovery duration, a larger displacement, and faster COP. However, when the magnitude of perturbation increases, whether older adults with CLBP adopt different movement strategies to regain their balance or simply accept higher risks of falls is uncertain.

In the current study, the CLBP group complained about having had back pain problem for at least 6 years, that it had a moderate effect on their physical functions, and that it resulted in a higher fear avoidance of physical activity even though their present level of physical activity was not significantly different from that of the healthy participants. Studies have suggested that people who had more fear avoidance beliefs had more pain and disability, and also had a poorer prognosis [26–28]. Physical activity levels might also affect the prognosis of CLBP [61]. With aging and a possible deterioration of spinal functions, the pain, disability, and even physical activity in this specific population would expectedly get worse [62]. Therefore, early detection of any possible physiological limitations that might impede the functional independence of older adults with CLBP is critical. The present study showed the presence of balance deficits in this group when responding to an unexpected postural perturbation. However, whether higher fear avoidance beliefs of physical activity, or a lower level of physical activity is associated with the COP movements during reactive balance control in older adults with CLBP remains unclear.

This study has some limitations. Kinematics and electromyography of the lower extremities and trunk were not used in this study. Information about movement patterns would provide explanations for the observed differences in the COP in the present study, and the electromyography would examine the underlying mechanism for balance and neuromuscular control. Moreover, although the participants were instructed to pull the string only with their arms as firmly as they could, some weaker participants might have leaned backward to achieve the target pulling force and changed the posture before perturbation. Additional studies are needed to evaluate these problems.

The magnitude of the pulling force was determined based on our pilot testing of healthy older adults. The present study showed that the CLBP group had less grip strength than did the HEA group, which might imply that this type of pulling task poses a greater challenge to the arms and upper body of patients with CLBP. Participants in the CLBP group had a wide range of CLBP duration: from a few months to a few decades. It is possible that people who have a longer history of pain also have greater balance impairments. Finally, our study population was small and might lead to a higher type I error. Because of these considerations, care must be taken in interpreting our data and findings.

## Conclusion

Responding to a suddenly released pulling force, e.g., a stuck drawer or a heavy object, is commonly encountered during daily life. The present study showed that older adults with CLBP had poorer postural responses—delayed reaction, larger displacement, higher velocity, longer path length and greater sway area of COP─than did healthy older adults when responding to s suddenly released pulling force. For the 4-kg pulling force, older adults with CLBP tended to lose their balance nonsignificantly more often than did older adults without CLBP. Both older adults with and without CLBP had larger postural responses in reaction to the 4-kg pulling force. The present study specified that the reactive balance deficits in the older adults with CLBP implying a greater risk of falls when the postural disturbance is unexpected and getting larger. It is commonly accepted that aging is associated with declines in balance and postural control. Our findings highlight the impact of CLBP on reactive balance in older adults when they respond to a suddenly released external pulling force. This study also suggests that for older adults with CLBP, in addition to managing their pain and disability, their reactive balance should be evaluated, and reaction and movement strategy training should be included in the intervention program. Because of their poorer reactive responses compared with healthy adults, both clinicians and older patients with CLBP should be advised about the significance of impaired reactive balance and risk of falls when encountering unexpected perturbations.

## Supporting Information

S1 Data Data for both older adults with and without CLBP. (XLSX)
